# A Double Payload Complex between Hypericin and All-*trans* Retinoic Acid in the β-Lactoglobulin Protein

**DOI:** 10.3390/antibiotics11020282

**Published:** 2022-02-21

**Authors:** Beatriz Rodríguez-Amigo, Cormac Hally, Núria Roig-Yanovsky, Pietro Delcanale, Stefania Abbruzzetti, Montserrat Agut, Cristiano Viappiani, Santi Nonell

**Affiliations:** 1 Institut Quimic de Sarrià, Universitat Ramon Llull, 08017 Barcelona, Spain; beatrizrodrigueza@iqs.url.edu (B.R.-A.); cormachallyg@iqs.url.edu (C.H.); nroigy@iqs.url.edu (N.R.-Y.); montserrat.agut@iqs.url.edu (M.A.); 2 Dipartimento di Scienze Matematiche, Fisiche e Informatiche, Università di Parma, 43124 Parma, Italy; pietro.delcanale@unipr.it (P.D.); stefania.abbruzzetti@unipr.it (S.A.)

**Keywords:** acne vulgaris, retinoic acid, hypericin, β-lactoglobulin, photodynamic therapy

## Abstract

Combined therapies are usually used to treat *acne vulgaris* since this approach can tackle various foci simultaneously. Using a combination of spectroscopic, computational, and microbiological techniques and methods, herein we report on the use of β-lactoglobulin as a double payload carrier of hypericin (an antimicrobial photodynamic agent) and all-*trans* retinoic acid (an anti-inflammatory drug) for *S. aureus* in vitro photodynamic inactivation. The addition of all-*trans* retinoic acid to hypericin-β-lactoglobulin complex renders a photochemically safe vehicle due to the photophysical quenching of hypericin, which recovers its photodynamic activity when in contact with bacteria. The ability of hypericin to photoinactivate *S. aureus* was not affected by retinoic acid. β-Lactoglobulin is a novel biocompatible and photochemically safe nanovehicle with strong potential for the treatment of acne.

## 1. Introduction

*Acne vulgaris*, or just acne, is a chronic inflammatory disease of the pilosebaceous unit-hair follicles in the skin associated with a sebaceous gland. It results from an altered keratinization, inflammation, and bacterial colonization of hair follicles by *Propionibacterium acnes*, while other bacterial strains present in the skin microbiota, such as *Staphylococcus aureus*, may also be involved [1,2].

Around 630 million people worldwide suffer from this condition, mostly teenagers, which can be painful and frequently cause significant embarrassment and anxiety in affected patients [3]. *Acne vulgaris* accumulates on areas with a greater number of sebaceous glands, such as the face, neck, chest, upper arms and back, causing non-inflammatory injuries (comedones) or inflammatory injuries (papules, pustules and large red bumps) which may end up in various degrees of scarring [1,4].

Several approaches, both topical and systemic, are attempted to overcome *acne vulgaris*. Systemic oral contraceptive pills can be used to reduce androgen-levels causing a reduction in sebum production [1], while retinoids, namely all-*trans* retinoic acid (tretinoin, RA) and 13-*cis* retinoic acid (isotretinoin) are used to diminish inflammation and normalize the desquamation of the follicular epithelium [5,6]. A third approach regarding *acne vulgaris* is the antimicrobial one, in which benzoyl peroxide and antibiotics, such as clindamycin and erythromycin, are used to disinfect skin afflicted by *acne vulgaris*. Typically, combination therapies with different type of drugs are used to treat acne [1,4].

Following current trends, microbial resistance to antibiotics has also been detected in acne and therefore alternatives to these treatments must be developed [7,8]. In this regard, photodynamic therapy (PDT) is a promising alternative to antibiotics based on the combination of a photosensitizer, light and molecular oxygen. These three elements, which are devoid of any toxicity per se, generate reactive oxygen species that cause oxidative damage to cells resulting in their inactivation [9]. PDT has been already attempted in vitro and in vivo successfully in acne treatments either alone or in combination with antibiotics [10,11,12,13,14].

Many active ingredients in pharmaceuticals are insoluble in aqueous solutions and need a vehicle to be transported to the adequate site for action. Several strategies have been tried, such as derivatization or the use of nanoparticles, liposomes, proteins, among other carriers. Regarding proteins, it is known that β-lactoglobulin (βLG), a homodimeric protein belonging to the lipocalin family, presents a remarkable versatility in their ligand-binding patterns and is known to accommodate hydrophobic molecules like hypericin, cholesterol, vitamin D, retinoids, or linear fatty acids in its structure [15,16,17]. 

**Figure 1:**
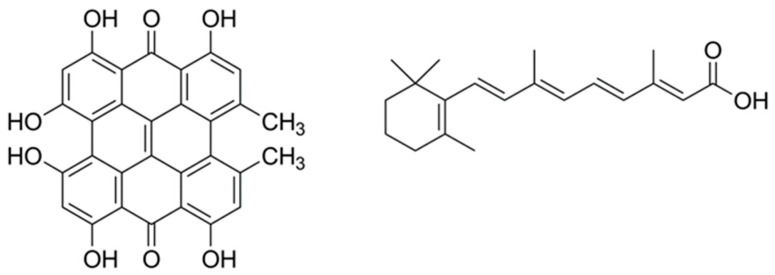
The molecular structures of hypericin (**left**) and all-*trans* retinoic acid (**right**).

Of particular interest for this study, we have recently shown that hypericin (Hyp), a naturally occurring photosensitizer extracted from the plant *Hypericum perforatum*, shows photoantimicrobial properties when delivered to bacteria using βLG [17]. Hyp has also been shown to decrease the population of *P. acnes* through PDT [18]. In this study, we report the characterization and antimicrobial activity of a βLG complex with a double payload of Hyp and RA to assess its potential as a combined agent for the treatment of acne. Figure 1 presents the molecular structures of Hyp and RA.

## 2. Results

### 2.1. Interaction between Retinoic Acid and Monomeric β-Lactoglobulin

**Figure 2:**
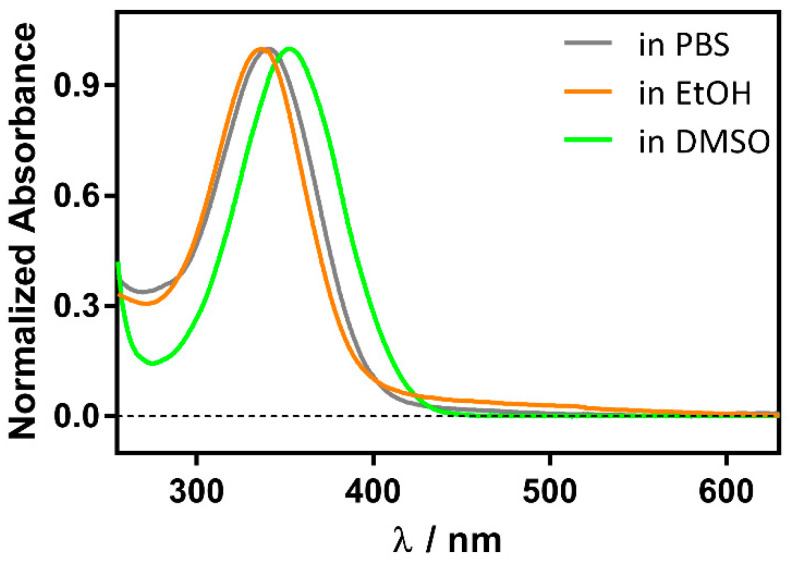
Solvent polarity effect on the normalized absorption spectrum of 1 μM RA.

Although RA is more stable than the *cis* isomer, it degrades under daylight within minutes to a few hours, depending on the solvent and temperature, due to oxidation of the conjugated double bonds, resulting in a complex mixture of products (Figure S1). However, in the presence of βLG it is stable over several hours, as previously observed for retinol [19]. Moreover, the RA UV spectrum shifts from 336 nm in phosphate buffer saline (PBS) to 350 nm in the presence of βLG. For comparison, a similar redshift is observed in aprotic solvents such as dimethylsulfoxide (DMSO), where RA absorbs at 353 nm (Figure 2). Taken together, these observations suggest the formation of a RA-βLG complex where RA is partially or totally shielded from the aqueous environment.

**Figure 3:**
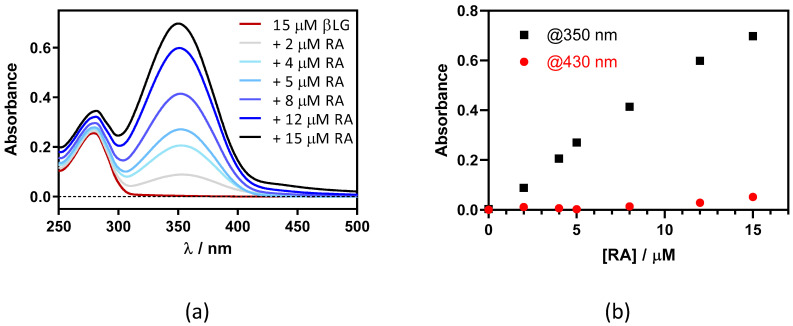
(**a**) Absorption spectra of retinoic acid (RA)–β-lactoglobulin (βLG) mixtures at increasing RA concentrations (between 0 and 15 μM). βLG concentration was fixed at 15 μM. (**b**) The evolution of absorbance at 350 nm and 430 nm upon titration.

This was confirmed by adding RA to a fixed concentration of βLG (15 μM) in PBS (Figure 3). Two maxima can be observed in the absorption spectrum, one at 280 nm and the other at ~350 nm, which are assigned to βLG and RA, respectively (Figure 3a). At RA concentrations higher than 5 μM, a new band at 430 nm appears due to the formation of RA aggregates in solution. As can be observed in Figure 3b, the band at 350 nm linearly increases upon addition of the RA, while the band at 430 nm only begins to increase above 5 μM of RA. This indicates that RA only starts to aggregate when the protein is saturated by RA. 

**Figure 4:**
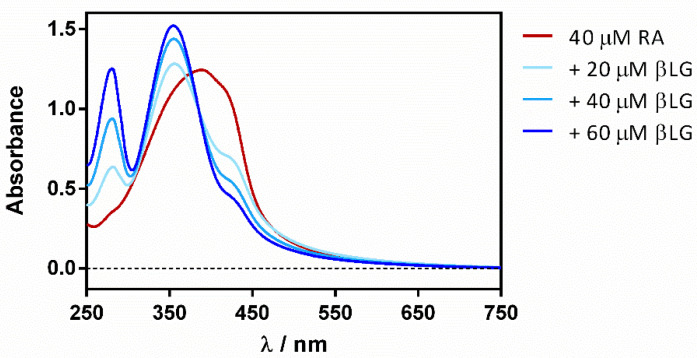
βLG addition effect on the absorption spectrum of free RA in PBS.

Reverse titration (adding the protein to the ligand) showed a gradual deaggregation of RA in a water solution, shifting the initially non-structured band at 430 nm to a well-structured band at 355 nm (Figure 4).

**Figure 5:**
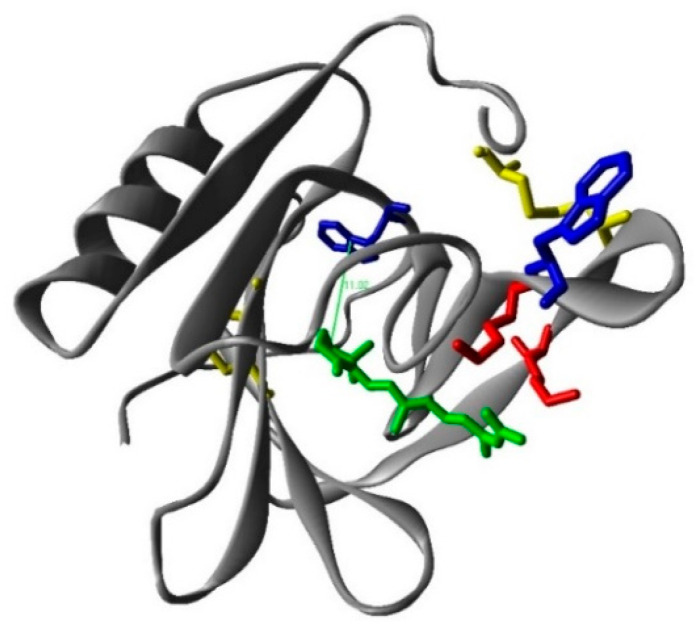
The calculated structure of the β-lactoglobulin (monomer)–retinoic acid complex. Red: lysines; blue: tryoptohan 19 (back) and 61 (front); yellow: cysteines; green: retinoic acid.

To prove the binding of RA to βLG, fluorescence quenching was measured. Based on spectral overlap, Förster resonance energy transfer (FRET) is expected to occur between tryptophan (Trp-) residues as donors and the RA ligand as the acceptor [20]. The βLG monomer contains two fluorescent Trp residues at positions 19 and 61, in which Trp19 is in an apolar environment within the central hydrophobic β-barrel or calix, while Trp61 is part of an external loop, partly exposed, and its fluorescence might be partially quenched by the proximity of the Cys66–Cys160 disulphide bond (Figure 5) [21]. Therefore, it is possible to use FRET from the intrinsic Trp fluorescence (Trp61 and Trp19) in βLG as a tool to study the interaction of RA with the protein. 

**Figure 6:**
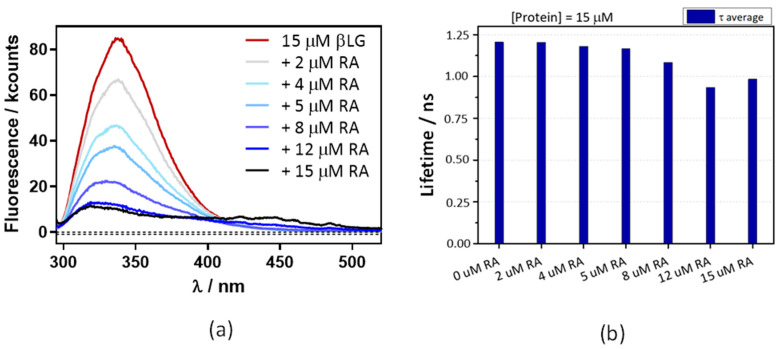
A decrease in βLG fluorescence lifetime (**a**) and intensity (**b**) with increasing concentrations of RA. βLG concentration was fixed at 15 μM, excitation at 280 nm, and detection at 340 nm. The fluorescence showed multiexponential decay, thus the amplitude-averaged decay lifetime is plotted.

Figure 6a shows that the βLG fluorescence intensity strongly decreases in the presence of RA, confirming the interaction between RA and βLG. The fluorescence lifetime also shows a concentration-dependent decrease, albeit to a lesser extent (Figure 6b). This suggests that, in addition to FRET, another quenching mechanism is operating in the complex. From the lifetime data the energy transfer efficiency is calculated as 0.17. 

To gauge the validity of this interpretation, the Förster critical distance for energy transfer between Trp and RA (*R*_0_) was estimated by means of Equation (1) [22].

(1) $$R_{0}{}^{6}=\frac{9\cdot \ln\left(10\right)\cdot \kappa^{2}\cdot \Phi_{F}\cdot J}{128\cdot \pi^{5}\cdot n^{4}\cdot N},$$

where κ is the orientation factor for the dipole–dipole interaction, *Φ*_F_ is the fluorescent quantum yield of βLG in the absence of RA, *J* is the spectral overlap integral between the fluorescence spectra of βLG and the molar absorption coefficients of RA, *n* is the refractive index of the medium between the Förster pair, and *N* is Avogadro’s constant. The refractive index of the protein βLG is *n* = 1.594 [23], the fluorescent quantum yield was measured by comparison using tryptophan as a reference (*Φ*_F_ = 0.04), the orientation factor was assumed to be 2/3, and the spectral overlap integral was calculated using Equation (2) [22]:

(2) $$J=\frac{\int F_{d}\left(\lambda \right)\cdot \varepsilon_{a}\left(\lambda \right)\cdot \lambda^{4}\cdot d\lambda }{\int F_{d}\left(\lambda \right)\cdot d\lambda },$$

where *F_d_*(λ) is the emission spectrum of the donor normalized to an area of 1 and *ε_a_*(λ) the absorption coefficient spectrum of the acceptor. Using these values, *R*_0_ = 7.7 Å is obtained for the Trp-RA pair. Since the energy transfer efficiency is *E =* 0.17, the distance between Trp19 and RA in βLG can be estimated as *R* = 10 Å using Equation (3), in excellent agreement with the 11 Å found computationally between Trp19 and RA (Figure 5).

(3) $$E=\frac{R_{0}{}^{6}}{R_{0}{}^{6}+R_{}^{6}}.$$

### 2.2. Interaction between Retinoic Acid and Dimeric β-Lactoglobulin

**Figure 7:**
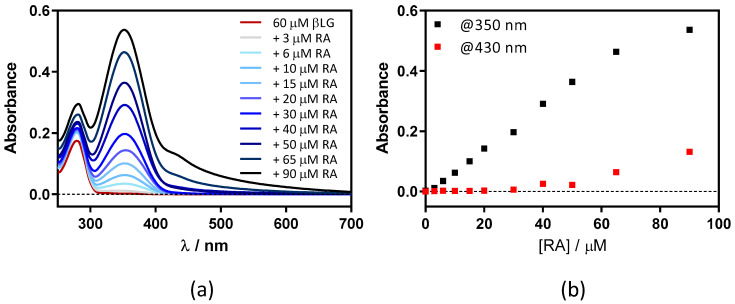
(**a**) Absorption spectra of retinoic acid (RA)–β-lactoglobulin (βLG) mixtures at increasing RA concentrations (between 0 and 90 μM). βLG concentration was fixed at 60 μM. (**b**) The evolution of the absorbance at 350 nm and 430 nm upon titration.

**Figure 8:**
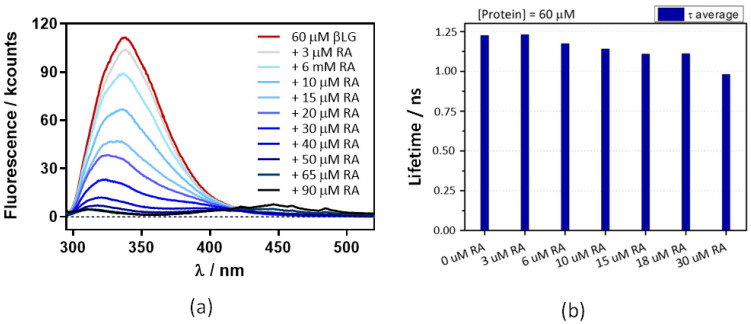
Decreases in β-lactoglobulin (βLG) fluorescence intensity (**a**) and lifetime (**b**) with increasing concentrations of retinoic acid (RA). The βLG concentration fixed at 60 μM, excitation was at 280 nm, and detection at 340 nm. The fluorescence showed a multiexponential decay, thus the amplitude-averaged decay lifetime is plotted.

The above experiments were repeated at 60 μM βLG, where the protein is in its dimeric form, which is essential for binding Hyp [17]. Absorbance (Figure 7) and fluorescence (Figure 8) results were very similar, indicating that neither RA binding to the protein nor fluorescence quenching were affected by protein dimerization.

### 2.3. Spectroscopic Characterization of the Ternary Complex between β-Lactoglobulin, Retinoic-Acid, and Hypericin

Since Hyp only binds to the pocket formed by dimerized βLG [17], protein concentrations above 40 μM were used to ensure that the protein was found in its dimeric form. Different addition orders of the three components to the formulation proved no substantial differences neither in absorption nor in fluorescence, indicating that, regardless of the order of addition, the complex seems to have the same properties (Figure S2).

Figure 7 shows the absorption and fluorescence plots resulting from titrating the βLG–Hyp complex with RA. The experiment yielded similar results irrespective of Hyp concentration (1, 4, or 8 µM).

**Figure 9:**
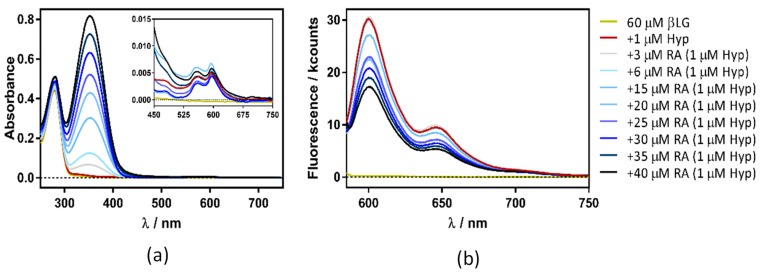
Absorption (**a**) and fluorescence emission (**b**) spectra of hypericin (Hyp)-retinoic acid (RA)–β-lactoglobulin (βLG) at increasing RA concentrations (between 0 and 50 μM). The concentration of βLG and Hyp were 60 μM and 1 μM, respectively. Emission spectra were collected exciting the samples at 554 nm.

**Figure 10:**
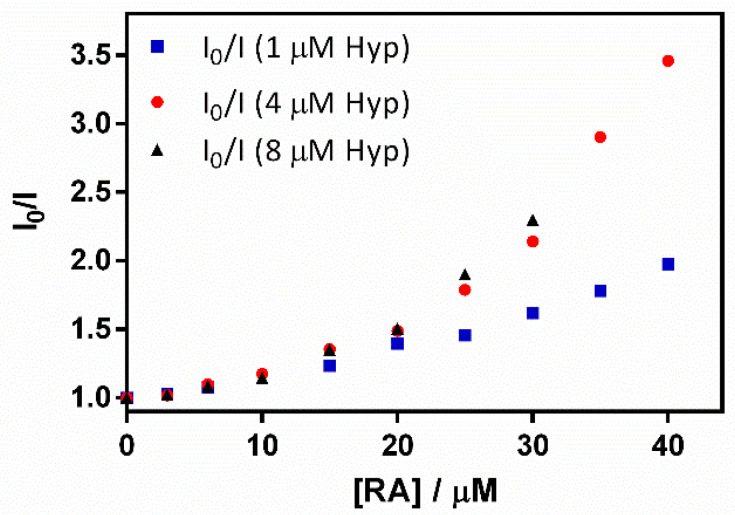
A Stern–Volmer plot for the quenching of the hypericin (Hyp) fluorescence–retinoic acid (RA)–β-lactoglobulin (βLG) complex (fixing βLG concentration at 60 µM) with different Hyp concentrations (1, 4, and 8 μM) upon the addition of increasing RA concentrations.

Surprisingly, in addition to the expected increase in absorbance due to RA (Figure 9a), a gradual decrease in Hyp’s fluorescence (excited at 554 nm) was observed (Figure 9b). To elucidate the process taking place, fluorescence quenching at three different Hyp concentrations was analyzed by the aid of Stern-Volmer plots (Figure 10).

The plots presented an initial linear trend until about 20 μM of RA, after which an upward curvature begins to take place, which is much more evident for 4 and 8 μM than for the lower Hyp concentration. This non-linear relationship suggests a concentration-dependent competition between dynamic and static quenching [24]. 

Stern-Volmer plots for dynamic quenching (Equation (4)) and for simultaneous static and dynamic quenching (Equation (5)) were fitted to the data obtained [22].

(4) $$\frac{I_{o}}{I}=1+K_{D}\cdot \left[\mathrm{RA}\right]$$

(5) $$\frac{I_{o}}{I}=1+\left(K_{D}+K_{s}\right)\cdot \left[\mathrm{RA}\right]+K_{D}\cdot K_{S}\cdot {\left[\mathrm{RA}\right]}^{2}$$

*K_D_* was fitted to the initial part of the series by means of Equation (4), obtaining Stern-Volmer constants that did not depend on Hyp concentration (*K_D_* = 2.3 × 10^4^ M^−1^, 2.4 × 10^4^ M^−1^, and 2.3 × 10^4^ M^−1^ for 1, 4 and 8 μM Hyp, respectively). On the other hand, *K*_S_ was fitted with Equation (5), determining values of 1.6 × 10^4^ M^−1^, 4.9 × 10^4^ M^−1^, and 4.6 × 10^4^ M^−1^ for 1, 4 and 8 μM Hyp, respectively. The increase in *K*_S_ at higher Hyp concentrations is consistent with the binding of two Hyp molecules by the βLG dimer at high Hyp concentrations [17].

**Figure 11:**
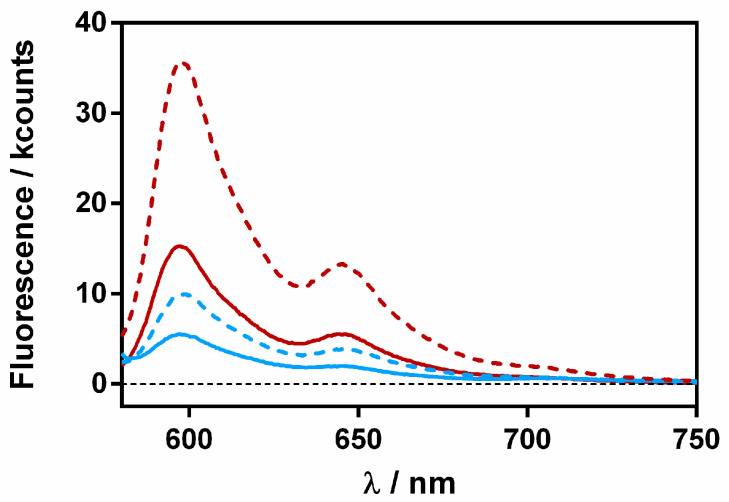
Fluorescence emission spectra of βLG-Hyp complex (60 μM and 8 μM, respectively) in the presence of 20 (red) and 40 (blue) μM RA. The solid lines represent the complex in solution and the dashed ones after a 30-min incubation with *S. aureus*.

A final study was performed to assess whether Hyp and RA separate upon binding to the bacteria cells. We reasoned that upon delivery to the cells, spatial separation between Hyp and RA would restore the fluorescence of Hyp. The fluorescence of Hyp-RA-βLG complexes containing 20 and 40 μM RA was recorded in the absence and presence of *S. aureus* bacteria (Figure 11).

For both complexes, fluorescence increased in the presence of the bacterial cells, confirming the separation between Hyp and RA. However, it is worth noting that the final fluorescence intensity was not the same for both complexes, indicating that a fraction of the Hyp and RA molecules were still close together, probably within the protein.

The quenching of the triplet excited state of Hyp by RA was also studied by laser flash photolysis. Figure S4 shows the time-resolved triplet absorption signals of the Hyp-βLG complexes in the presence of different amounts of RA. In the absence of bacteria (Figure S4a) the effect of RA is to quench the amplitude, but not the lifetime, of the signal, consistent with the quenching of Hyp’s singlet state, the precursor of the triplet. In the presence of *S. aureus* (Figure S4b) there is a partial recovery in the triplet signal, indicating the separation of Hyp and RA, albeit not complete. At the highest RA concentration, the Hyp triplet state is completely quenched despite the presence of bacteria.

Taken together, the above results reveal that the excited states of Hyp are quenched by RA while they are closely located, namely within the protein. This indicates that the Hyp-RA-βLG complex is a photochemically safe vehicle thanks to the RA-induced silencing of the photophysical properties of hypericin. 

### 2.4. Antimicrobial Studies

After having established the quenching of the hypericin excited states in the ternary complex and their recovery in the presence of bacteria, we performed photoinactivations to study the effects of the ternary complex on *S. aureus*, which is involved in *acne vulgaris* and also in furunculosis and cellulitis [25]. Photoinactivation of *P. acnes*, as the main actor in acne pathogenesis, was ruled out since it needs anaerobic growth conditions [26], under which PDT in vitro experiments could not be performed.

Prior to using the ternary complex to inactivate the bacteria, the individual components were tested independently and in combination with others. βLG and RA did not present any cytotoxic activity in the dark nor under green light illumination at concentrations up to 100 μM for the protein and 10 μM for RA. Furthermore, the dual complex βLG-RA did not cause significant changes in the bacterial growth, neither in the dark nor under 18 and 37 J·cm^−2^ green light fluence (Figure S5).

**Figure 12:**
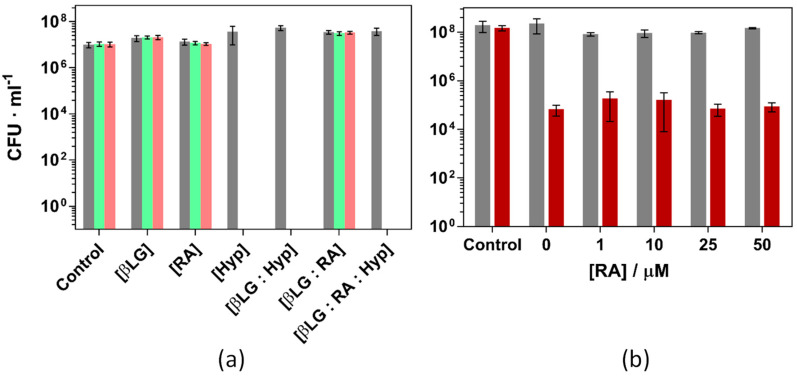
Photoinactivation of *S. aureus* ATCC 29213 under green light (521 ± 19 nm) and (**a**): Combinations of βLG, RA and Hyp (40, 10 and 4 μM, respectively) exposed to 18 (green) and 37 (pink) J·cm^−2^ of light fluence. (**b**): Combinations of βLG (40 μM), Hyp (1 μM), and increasing concentrations of RA, exposed to 5 J·cm^−2^ of light fluence (red bars). Grey bars are the dark controls in both panels.

Figure 12 presents the inactivation of *S. aureus* under different experimental conditions. Figure 12a shows the effect of each individual component and their binary and ternary combinations using βLG, RA and Hyp concentrations of 40 μM, 10 μM, and 4 μM, respectively and exposing the cultures to 18 and 37 J·cm^−2^ of green light. Only illuminated formulations which contained Hyp were able to completely inactivate the bacterial strain. Any other condition, light or dark, did not induce any cell death whatsoever. The results are in agreement with previous studies performed with the binary complex βLG:Hyp [17]. Hyp 4 μM induces a complete inactivation of the antimicrobial strain and RA has no effect on the outcome of the treatment. 

RA, thanks to the polyene chain in its structure, can efficiently scavenge ROS. In this regard, there was a concern during the design of the study about a possible reduction of the bacterial inactivation due to the introduction of a singlet oxygen scavenger such as RA. Since Hyp may oxidize RA and then inactivate the strain, Figure 12b presents the results of *S. aureus* the photoinactivation studies were repeated under milder conditions (1 μM Hyp, 40 μM βLG and 5 J·cm^−2^ light fluence, causing a reduction of 3-log units). Addition of RA up to 50 μM caused no effect on the outcome of the experiment.

An uptake assay was performed to assess the extent of hypericin binding to the bacteria. After the 30-min incubation period with the ternary formulation, the solution was centrifuged three times and the pellet resuspended in fresh PBS, before finally being dissolved in DMSO to lyse the bacteria and monomerize the photosensitizer. Fluorescence spectra were acquired before each step to monitor the location of Hyp during photosensitization (Figure S6). The fluorescence spectrum acquired immediately after incubation presented the typical unaggregated emission of Hyp due to its binding to the protein. In sharp contrast, most of the fluorescence was lost after the first centrifugation, indicating the removal of βLG from the supernatant and either removal or aggregation of Hyp onto the bacterial cells. Finally, fluorescence was recovered once the cells were lysed in DMSO and the photosensitizer was monomerized once again in solution. 

Hyp uptake by *S. aureus* from formulations with increasing concentrations of RA was quantified by spectrofluorimetry, interpolating the fluorescence intensity in a calibration curve (Figure S7). Regardless of the RA concentration, the uptake of Hyp by *S. aureus* resulted in 0.6 μM from an initial nominal 4 μM, indicating that only 15% of the photosensitizer was bound to the bacteria. The addition of RA, with concentrations ranging from 0 to 50 μM, did not influence Hyp uptake by *S. aureus*.

## 3. Discussion

Our results show that Hyp, RA and βLG form a ternary complex, the structure and stoichiometry of which depends on the concentrations of the two ligands. RA quenches the fluorescence emission from both βLG and Hyp, the first through a combination of a FRET mechanism and static quenching, while the latter is via collisional dynamic quenching at low RA concentrations, in addition to static quenching at higher concentrations. Quenching of the singlet state precludes the population of the longer-lived triplet state and hence the production of singlet oxygen and its photodynamic effects. The quenching observed in the formulation does not inhibit the pharmacological activity of Hyp against *S. aureus* since the photophysical properties of the photosensitizer are recovered once in contact with bacteria. 

Some of the studies available in the literature suggest that RA is bound to an external surface cleft between the β-barrel and the α-helix, in which Phe136 and Lys141 stabilize the interaction [27]. However other modelling calculation studies confirmed that Trp19 is the one involved in the energy transfer analysis, which is deep inside of the calyx and can then interact with Lys70 [28]. Moreover, a nice study by Cho et al. supports this last theory [29]. These authors prepared four different mutants replacing the four amino acids, which play a role in the interaction with RA. The mutant that shows a marked decrease in its binding is the one lacking the Lys70, which allowed to conclude that RA binding must be within the calyx. The three-dimensional structures of the complexes between βLG and RA, and retinol determined with X-ray crystallography conclusively demonstrated that binding occurs at the calyx. Our FRET quenching results are consistent with this conclusion. Importantly, binding geometry is very similar for retinol and RA (Figure S3) [30]. 

Various published studies which also pursue the goal of treating acne with PDT have proved its feasibility in vitro, in vivo and in clinical trials. De Annunzio and co-workers used methylene blue, curcumin and chlorin Ce6 for the inactivation of *P. acnes*, for which 3.3 J·cm^−2^ and 2.6 μM of Ce6 were required to fully inactivate the strain [10]. Another study using Ce6 published by Jeon and co-workers presents susceptibility of *P. acnes*, despite achieving only 2-log cell death and presenting dark toxicity, at 0.8 μM [31]. A later study by the same group proves also an anti-inflammatory effect of Ce6 in HaCaT cells, showing that one compound can treat both inflammation and infection at the same time [11]. Another Korean study proposed a dual therapy treatment for acne using lipase-sensitive liposomes to deliver the antibiotic erythromycin and the photosensitiser pheophorbide A to obtain an enhanced effect on *P. acnes* infections in vitro and in vivo [13]. Similarly, Xu and co-workers tested in clinical trials a combination of the antibiotic minocycline and the photosensitiser prodrug 5-aminolevulinic acid in “moderate to severe facial acne”, in which they saw an improvement of the clinical outcome and in quality of life of the patients when using the combined therapy in comparison with the antibiotic alone [14]. On the other hand, RA has been recently combined with PDT for the treatment of chromoblastomycosis [32]. 

The study reported herein suggests a new therapeutic option for acne, namely the simultaneous delivery of the anti-inflammatory agent RA with Hyp PDT using βLG as a biocompatible nanocarrier. We have shown that βLG dimers can host and transport Hyp and RA and that the nanoconstruct is photodynamically inactive, and therefore safe, due to quenching of the excited states of Hyp by RA. After Hyp delivery to the bacteria, the photodynamic activity is recovered, and bacteria are efficiently inactivated under exposure to green light. 

## 4. Materials and Methods 

### 4.1. Chemicals

Hyp was purchased from HWI Analytik GmbH, Ruelzheim, Gernamy. β-Lactoglobulin (isoform B) from bovine milk, retinoic acid (tretinoin) powder and Dulbecco’s phosphate-buffered saline (PBS pH = 7.4) were obtained from Sigma-Aldrich Chemical Co. (St. Louis, MO, USA). All other chemicals were commercially available reagents of at least analytical grade. Milli-Q water (Millipore Corporation, Bedford, MA, USA), with resistivity of 18 MΩ cm, was used. Once the complex PS-protein was prepared it was stored in the fridge and kept in the dark. For the microbiological cultures Tryptic Soy Broth and the agar-agar were also acquired from Sigma-Aldrich Chemical Co. (St. Louis, MO, USA).

### 4.2. Spectroscopic Techniques and Computational Chemistry

Absorption spectra were recorded on a double beam Cary 6000i UV-Vis-NIR spectrophotometer (Agilent Technologies, Santa Clara, CA, USA). Fluorescence spectra were recorded in a Fluoromax 4 spectrofluorometer (Horiba Jobin Yvon, Edison, NJ, USA).

Time-resolved fluorescence decays were recorded at a specific wavelength, selected by a monochromator grating, using a time-correlated single photon counting system (Fluotime 200, PicoQuant GmbH, Berlin, Germany) with a pulsed picosecond LED source for excitation, emitting at 280 nm and working at 10 MHz repetition rate. Decays were analyzed using the PicoQuant FluoFit 4.5.3 data analysis software. Absorbance of the samples were kept below 0.1 at the excitation wavelength in all cases and the photon counting rate was kept below 1%. The instruments’s response function (IRF) was measured using a suspension of Ludox® in water. Transient absorption experiments in the UV–visible (UV–vis) region were carried out using a home-built nanosecond laser flash photolysis system described elsewhere [33].

Computational chemistry visualization and calculations were performed using ViewerLite 4.2 from Biovia, San Diego, CA, USA. Distances between amino acid side chains were estimated using the tool “distance” of the program.

### 4.3. Microbial Strains, Culture Conditions, and Photodynamic Inactivation Studies

*Staphylococcus aureus* ATCC 29213 was obtained from the American Type Culture Collection. Bacterial cells were grown in sterile Tryptic Soy Broth (TSB, Panreac 413820.1210, Castellar del Vallès, Spain) at 37 °C overnight, whilst the following day an aliquot of the bacteria was grown in fresh TSB until an optical density 0.3 at 600 nm, which corresponds approximately to 10^8^ colony forming units per milliliter (CFU/mL). Cells were then washed three times with sterile PBS by centrifuging and resuspending the pellet. They were then incubated in the dark with the different photosensitizing agents for 30 min at room temperature at 37 °C under slight orbital agitation. Spectroscopic measurements were performed immediately after the incubation period. Photoinactivation experiments were carried out after the incubation period by placing 300 μL of the incubated bacteria in 96-well plates. Irradiation was carried out illuminating from the top with a Photocare LED source (Sorisa, Sant Quirze del Vallès, Spain), emission wavelength 521 ± 19 nm, for during 6, 15 or 30 min (5, 18 and 37 J·cm^−2^, respectively) and serially diluted until 10^−6^ times the original concentration. The diluted samples were seeded on Tryptic Soy agar (Merck, Darmstadt, Germany) and the colony forming units (CFUs) were counted the following day after incubation in the dark at 37 °C. Experiments were carried out in triplicate.

## 5. Conclusions

To summarize, the simultaneous double payload of the natural photosensitizer hypericin, for the treatment of bacterial pathogens, and retinoic acid, for its anti-inflammatory and desquamation properties, in a novel biocompatible and photochemically safe nanovehicle is an elegant approach with strong potential for the treatment of acne.

## Data Availability

The data presented in this study are available in the article and Supplementary Materials.

## Supplementary Materials

The following are available online at https://www.mdpi.com/article/10.3390/antibiotics11020282/s1. Figure S1: Time evolution of the absorption spectrum of, Figure S2: Absorption spectra of Hyp-RA-βLG mixtures, Figure S3: Crystal structures of the complex between retinoic acid and β-lactoglobulin and between retinol and β-lactoglobulin, Figure S4: Triplet absorption decay of Hyp-βLG complexes in the absence (black) and presence (red) of *S. aureus*, Figure S5: Photoinactivation of *S. aureus*, Figure S6: Fluorescence spectra of *S. aureus* incubated with the ternary complex βLG-RA-Hyp, Figure S7: Uptake of hypericin by *S. aureus*.
