# Supplementary material for: A Double Payload Complex between Hypericin and All-trans Retinoic Acid in the β-Lactoglobulin Protein
Source: Antibiotics (Basel). 2022 Feb 21;11(2):282. doi: 10.3390/antibiotics11020282 (PMC8868348; doi:10.3390/antibiotics11020282)
Supplement: Supplementary file 1 [file antibiotics-11-00282-s001.zip › antibiotics-1560236-Supplmentary.pdf]

antibiotics

MDPI

Supporting Information

A Double Payload Complex between Hypericin and All-*trans* Retinoic Acid in the β-Lactoglobulin Protein

Beatriz Rodríguez-Amigo [1,†], Cormac Hally [1,2,†], Núria Roig-Yanovsky [1], Pietro Delcanale [2], Stefania Abbruzzetti [2], Montserrat Agut [1], Cristiano Viappiani [2,*] and Santi Nonell [1,*]

1 Institut Quimic de Sarrià, Universitat Ramon Llull, 08017 Barcelona, Spain; beatrizrodrigueza@iqs.url.edu (B.R.-A.); cormachallyg@iqs.url.edu (C.H.), nroigy@iqs.url.edu (N.R.-Y.), montserrat.agut@iqs.url.edu (M.A.)
2 Dipartimento di Scienze Matematiche, Fisiche e Informatiche, Università di Parma, 43124 Parma, Italy; pietro.delcanale@unipr.it (P.D.); stefania.abbruzzetti@unipr.it (S.A.)
* Correspondence: cristiano.viappiani@unipr.it (C.V.); santi.nonell@iqs.url.edu (S.N.)
† These authors contributed equally to this work.

Citation: Rodríguez-Amigo, B.; Hally, C.; Roig-Yanovsky, N.; Delcanale, P.; Abbruzzetti, S.; Agut, M.; Viappiani, C.; Nonell, S. A Double Payload Complex between Hypericin and All-*trans* Retinoic Acid in the β-Lactoglobulin Protein. *Antibiotics* **2022**, *11*, 282. https://doi.org/10.3390/antibiotics11020282

Academic Editor: Marina DellaGreca

Received: 31 December 2021
Accepted: 17 February 2022
Published: 21 February 2022

Publisher's Note: MDPI stays neutral with regard to jurisdictional claims in published maps and institutional affiliations.

Copyright: © 2022 by the authors. Licensee MDPI, Basel, Switzerland. This article is an open access article distributed under the terms and conditions of the Creative Commons Attribution (CC BY) license (https://creativecommons.org/licenses/by/4.0/).

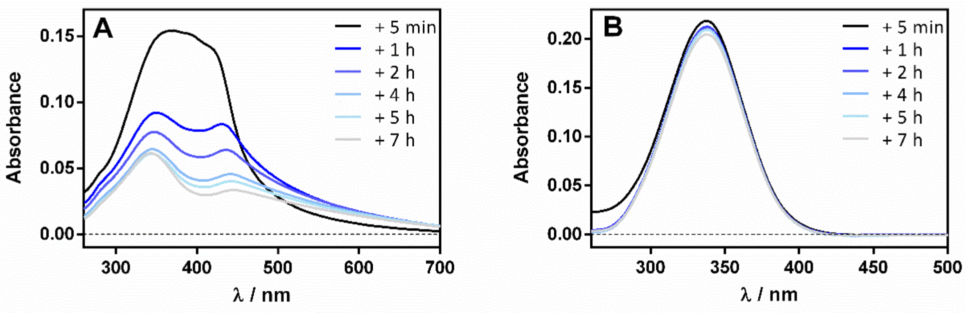

Figure S1. Time evolution of the absorption spectrum of 1.7 μM RA in A) water and B) EtOH.

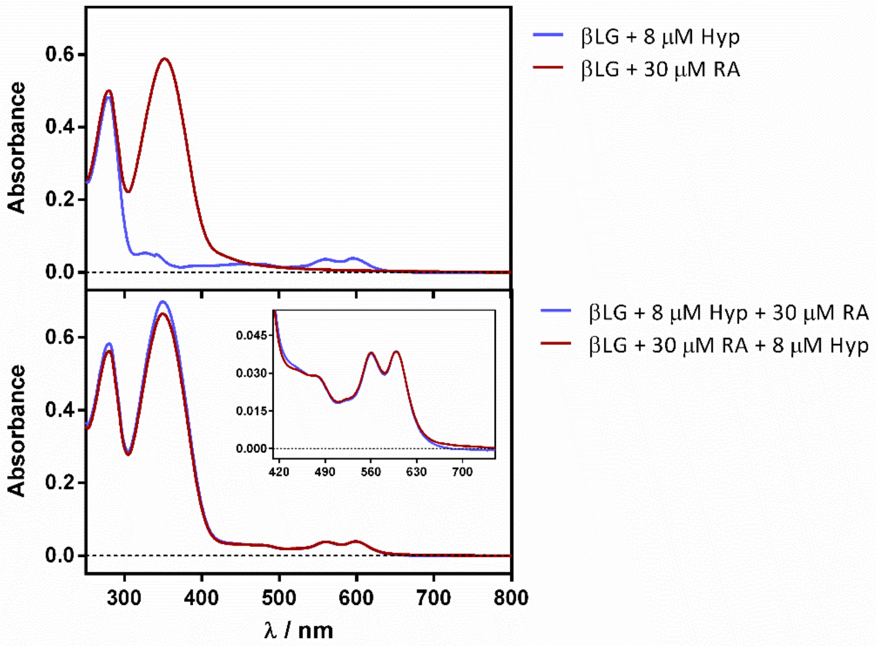

Figure S2. Absorption spectra of Hyp-RA-βLG mixtures, obtained after adding the components in

different order. 30 μM of RA (blue line) were added upon 60 μM of βLG and 8 μM of Hyp, and vice versa.

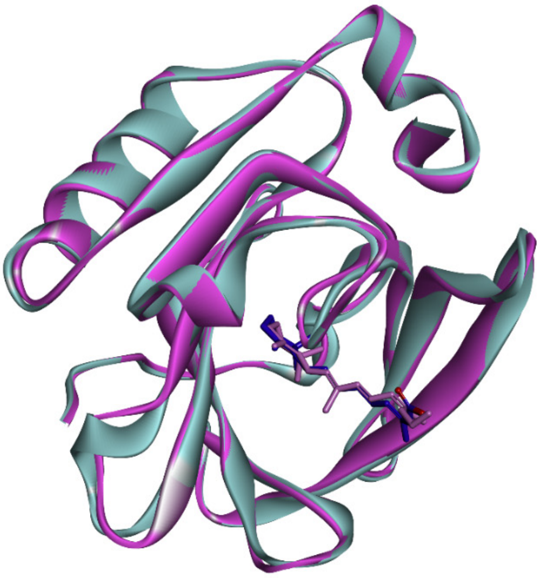

Figure S3. Crystal structures of the complex between retinoic acid and β-lactoglobulin (PDB 1GX9) and between retinol and β-lactoglobulin (PDB 1GX8). For 1GX9, the protein structure is represented as light blue solid ribbon, retinoic acid is represented as blue sticks. For 1GX8, the protein structure is represented as magenta solid ribbon, retinol is represented as magenta sticks.

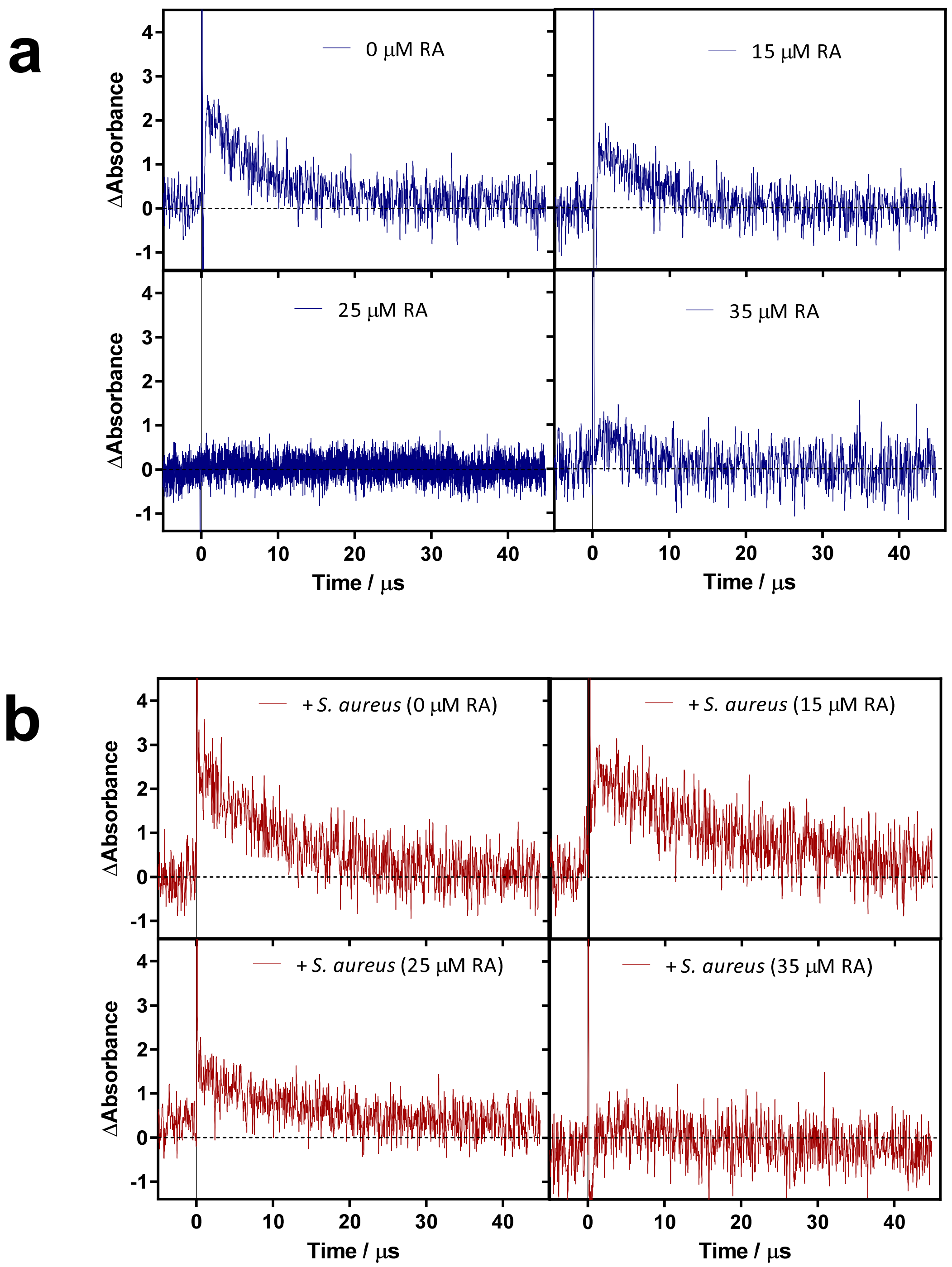

Figure S4. Triplet absorption decay of Hyp-βLG complexes in the absence (a) and presence (b) of *S. aureus*. Solutions containing 60 μM βLG and 15 μM Hyp and different RA concentrations were incubated with *S. aureus* cells for 30 minutes. Transients were acquired at 520 nm after pulsed-laser excitation at 532 nm averaging 100 shots.

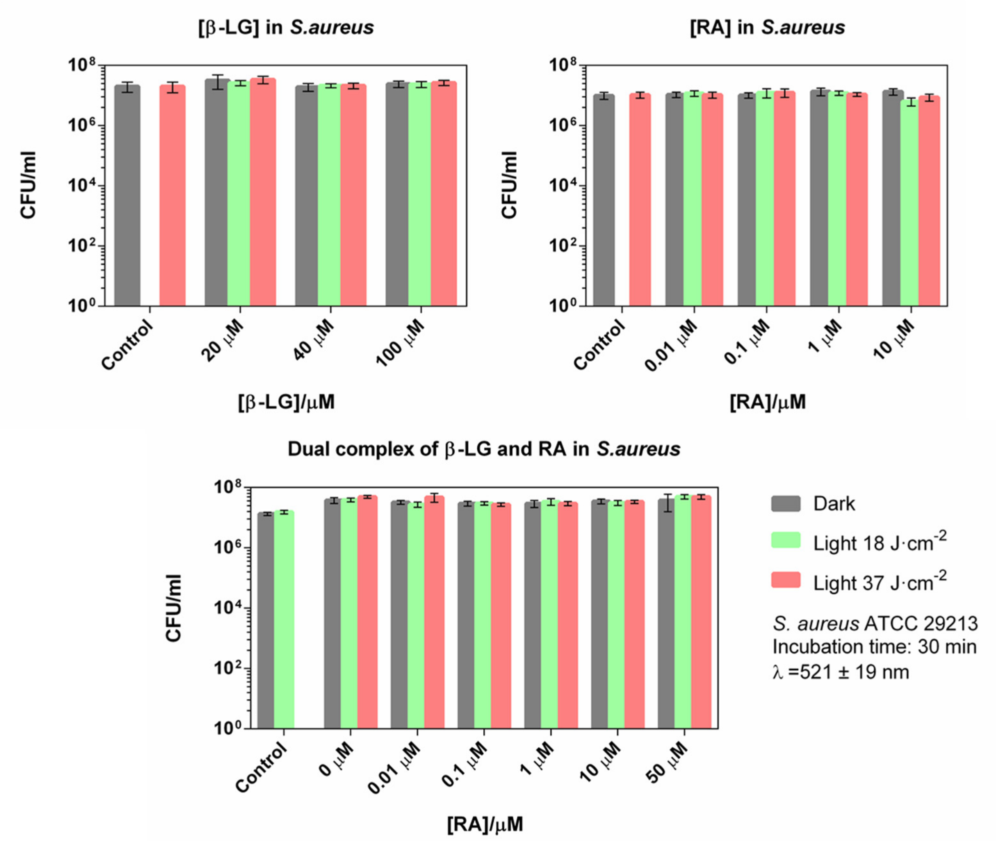

Figure S5. Photoinactivation of *S. aureus* with βLG (Top left), retinoic Acid (Top Right) and the dual complex βLG-RA (Bottom) using green light.

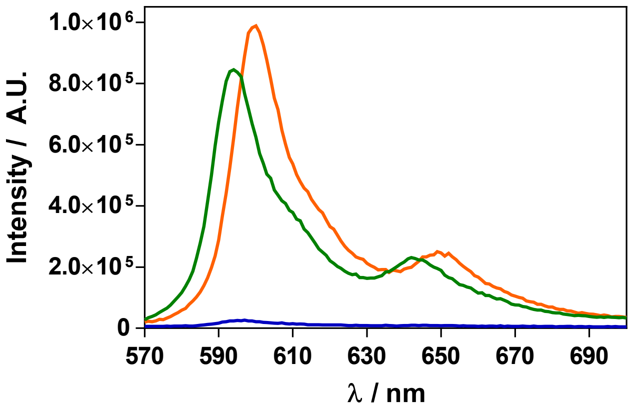

Figure S6. Fluorescence spectra of *S. aureus* incubated with the ternary complex βLG-RA-Hyp immediately after incubation without washing the supernatant (green), resuspending the pellet in PBS after the first centrifugation (blue) and the final pellet resuspended in DMSO after centrifugation (orange). Samples were excited at 550 nm.

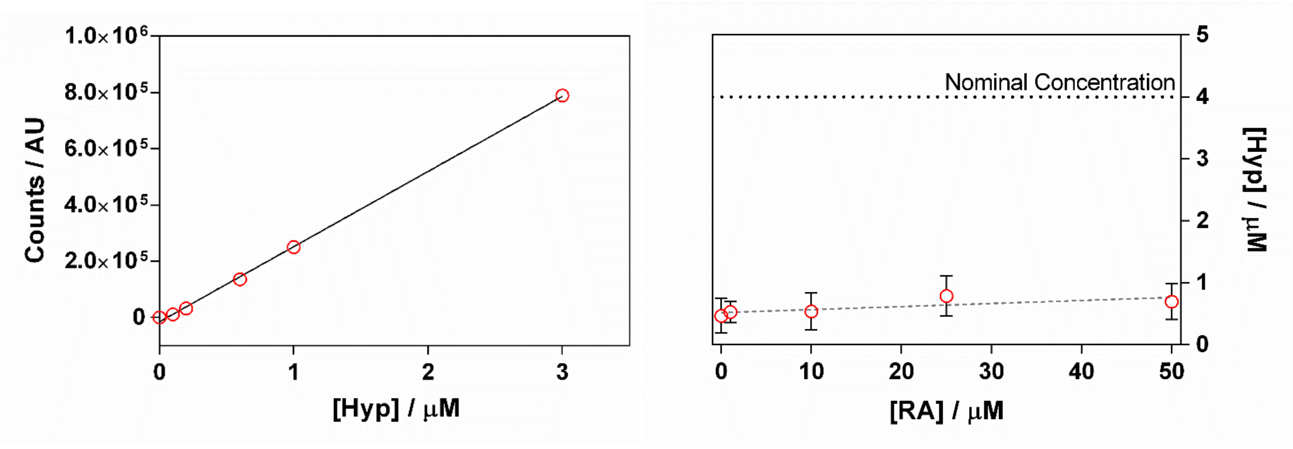

Figure S7. Uptake of hypericin by *S. aureus*, quantified by spectrofluorimetry (left), as a function of retinoic acid concentration (right).
